# Vaccines: From Empirical Development to Rational Design

**DOI:** 10.1371/journal.ppat.1003001

**Published:** 2012-11-08

**Authors:** Christine Rueckert, Carlos A. Guzmán

**Affiliations:** Department of Vaccinology and Applied Microbiology, Helmholtz Centre for Infection Research, Braunschweig, Germany; University of Alberta, Canada

## Abstract

Infectious diseases are responsible for an overwhelming number of deaths worldwide and their clinical management is often hampered by the emergence of multi-drug-resistant strains. Therefore, prevention through vaccination currently represents the best course of action to combat them. However, immune escape and evasion by pathogens often render vaccine development difficult. Furthermore, most currently available vaccines were empirically designed. In this review, we discuss why rational design of vaccines is not only desirable but also necessary. We introduce recent developments towards specifically tailored antigens, adjuvants, and delivery systems, and discuss the methodological gaps and lack of knowledge still hampering true rational vaccine design. Finally, we address the potential and limitations of different strategies and technologies for advancing vaccine development.

## Introduction

Scourges of humanity, such as smallpox, polio, and measles, have been controlled by vaccination. Other epidemics, for instance tuberculosis, have yet to be sufficiently restrained by immunization. Accordingly, policy makers have given a high priority to the development of novel vaccines to induce protective immunity against selected pathogens. Most human vaccines contain attenuated or killed pathogens and were developed empirically, such as the yellow fever vaccine [1], [2]. Safety concerns were associated with undefined vaccine preparations based on whole pathogens (e.g., inactivated or attenuated bacteria or viruses). Thus, novel subunit vaccines are based on a restricted number of individual components (i.e., antigens) of the specific pathogen, which are able to confer protective immunity. Obviously, the chances of finding effective components of subunit vaccines empirically are low. Immunogenic parts of pathogens that provide antigens for B cell receptors (BCRs) and antigenic peptides that are presentable by MHC molecules to T cell receptors (TCRs) have to be identified. It is critical to compensate for excluded pathogen-associated molecular patterns (PAMPs), which activate the innate immune system to induce an appropriate adaptive immune response. Finally, vaccine delivery systems may be needed. Hence, the rational design of vaccines is mandatory.

Rationally designed vaccines are composed of antigens, delivery systems, and often adjuvants that elicit predictable immune responses against specific epitopes to protect against a particular pathogen. In many cases a vaccine cannot be successfully designed due to insufficient knowledge about the mechanisms of protection. Although the repertoire of immune clearance mechanisms to fight pathogens is known, the specific contributions of different effector mechanisms are well-characterized for only a few pathogens. It is also largely unclear what determines the immunogenicity and selection of particular epitopes among all possible antigenic options offered by a pathogen. Which factors determine dominant or balanced immune responses? What are the mechanisms leading to long-term protection? Investigation of immune responses to known effective and ineffective vaccines and of pathogens' strategies of immune escape and evasion generates the basis to tackle these open questions. The approach relies on data from studies with empirically developed vaccines—for now and in the near future.

There are no universally accepted strategies and tools to rationally design vaccines. Vaccine development is still generally a tedious and costly empiric process. This review focuses on approaches to overcome empirical vaccine development and addresses their potential and limitations. It will become clear that even the latest developments are mostly first steps. Reports may sometimes sound too optimistic with regard to a prompt implementation of the introduced methods. Nevertheless, multiscale interdisciplinary efforts are strongly needed to reach this goal.

## Antigen Selection and Optimization

Selecting the optimal antigen represents the cornerstone in vaccine design. With the advent of genomics, the traditional process of selecting candidate antigens one by one has been replaced by reverse vaccinology approaches. Namely, the coding potential of a pathogen's genome is exploited by *in silico* selection, high throughput screenings, and profiling technologies (e.g., genomics, proteomics) to define promising antigens in relation to in vivo expressed genes and clonal variation [3]–[6]. Importantly, this approach is not suitable for nonproteinaceous antigens. Depending on the desired response, the antigenic protein should contain appropriate BCR epitopes and peptides that can be recognized by the TCR in a complex with MHC molecules. Synthetic peptides produced at comparably low cost can also be incorporated in subunit vaccines. This is relevant especially in epidemic situations when large amounts of vaccine doses need to be produced in a very limited period of time. A peptide-based vaccine meets high safety standards due to the possibility of excluding allergens, toxins, or other functional molecular domains of the pathogen. Restricting the immune response to defined antigenic regions can, furthermore, help avoid effects such as autoimmune responses, dominant responses against epitopes prone to antigenic drift, or responses against epitopes with specificity for a particular strain rather than multiple strains of the pathogen. However, the identification of immunogenic peptide sequences requires a considerable amount of experimental effort. Computational prediction methods can strongly reduce time and costs for vaccine development. Nevertheless, clonal variability and in vivo selection resulting in immune escape could render ineffective a vaccine based on short peptides encompassing a limited number of epitopes. Furthermore, there are technological constraints associated with this approach (e.g., synthesis of long polypeptides).

To elicit antibody responses, vaccines should include BCR epitopes. Their prediction is particularly challenging, though, and most B cell epitopes are discontinuous; that is, they are comprised of distant parts of the protein's primary structure. In addition, they are of variable length (3–30 amino acids) and conformation-dependent [7]. BCR epitopes do not possess physico-chemical patterns in their amino acid sequences that can be used for *in silico* prediction [8]. Some epitopes change conformation when interacting with the cognate antibody's paratope, making even 3-D structure-based prediction difficult [7]. The use of learning machines that depend on quantitative data on known antibody epitopes led to the development of prediction tools for linear epitopes such as BCPREDS [9], [10] and IMMUNOPRED [11], [12]. In contrast, PEPOP [13], [14] is based on 3-D structural data on antigen–antibody complexes, and it predicts discontinuous epitopes, their antigenicity, and immunogenicity, and suggests peptide constructs for synthesis. However, these methods have not yet reached sufficient predictive accuracy to be routinely applied in vaccine design.

The proteins or peptides of a subunit vaccine should also display sequences that allow T cell epitope formation in a complex with MHC molecules. MHC class I and II come in hundreds of alleles that are differentially combined between individuals. Choosing immunogenic peptides presented by MHC faces the challenge of not only predicting sequences appropriate for complexing with a particular MHC allele but also finding peptides that can reliably build epitopes in the diverse genetic background within a human population. Drawbacks of in vitro assay-based TCR epitope identification are (i) time consuming procedures for combinatorial coverage of relevant MHC alleles and candidate pathogenic antigens, (ii) high costs for peptide synthesis and reagents, and (iii) limited sensitivity when using naïve T cell populations. These efforts can be reduced extremely when combined with computational TCR epitope prediction [15], [16].

*In silico* prediction of T cell epitopes cannot be based on physico-chemical properties of presented peptides but depends on the application of learning machines on data sets of known MHC allele–peptide pairs. The development and maintenance of databases is absolutely essential to constantly improve predictions [17], [18]. Examples for such databases are IEDB [19], [20] or SYFPEITHI, which only lists experimentally validated natural MHC–peptide complexes [21], [22]. The tools OptiTope [23], [24] and NetMHCcons [25], [26] select for epitope peptides from specific MHC alleles or sets of MHC alleles as they occur naturally in individuals of a certain population. This is achieved by choosing promiscuous peptides that can be presented by several different MHC alleles of a supertype (i.e., universal peptides presented by most known alleles or a mixture of peptides binding to the most prevalent alleles within a population). The final goal is to provide suitable tools to generate immunogenic peptide sequences from any input antigen sequences. However, the broad applicability of these approaches towards rational vaccine design still remains to be proven.

Diversity also occurs at the level of the antigen. Immune escape of pathogen variants through mutation of immunogenic sequences has to be considered when selecting or designing antigens [27]. *In silico* generation of mosaic polyvalent antigens tackles this problem [28]. Immunization experiments with primates demonstrated the advantage of mosaic constructs over consensus or natural sequences to elicit T cell responses covering a broad selection of viral clades as well as antigenic immune escape variants that may evolve [29], [30]. The repertoire of possible immunogens can also be widened by exploring glycan antigens [31]. Whenever constructs are designed, one has to ensure their stability and thus bioavailability. For example, HIV-derived peptides display quite variable half-lifes in the cytosol of human cells and this has an impact on their recognition by CD8^+^ cells [32]. Structural vaccinology is a powerful emerging approach to optimize immunogens based on atomic-level structural information on requirements for conferring protective immunity [33]–[35]. Upon identification of immunogenic domains, it is possible to design constructs that lack decoy or masking portions of the antigen, such as epitope scaffolds that are able to elicit antibody responses against otherwise immune-recessive, cryptic, or transient epitopes [36]. It is also possible to engineer an optimized structure to enable broadly cross-protective responses. As example, chimeric proteins to effectively vaccinate against group B streptococci or *Neisseria meningitidis* were generated [37], [38].

## Adjuvants

Subunit vaccines are likely to lack the molecular cues needed for efficient activation of the innate immune system, thereby failing to induce vigorous adaptive immunity. PAMPs can act as adjuvants, however many pathogen-derived products might exhibit toxic activity [39]. The only globally approved adjuvant for humans is alum. It facilitates T_H_2-dependent immune responses but promotes less effective cytotoxic responses and can cause side effects. A number of other adjuvants have been recently approved for use in defined human vaccines, such as MF59 and monophosphoryl lipid A-containing formulations [40], [41], and there are other candidates in the pipeline. Adjuvants are not licensed per se, but as part of vaccine formulations. This together with stringent requirements for reagents used on healthy individuals raise the costs of clinical development [41]. Considerable effort was invested in the development of adjuvants for mucosal immunization [42]. Vaccination via mucosal routes is known to elicit both mucosal and systemic immunity [43], fighting pathogens at the site of entry. However, safety issues were observed following intranasal vaccination with the heat labile toxin of *Escherichia coli* and its attenuated derivative [42], [44]. This will need to be considered for current candidate mucosal adjuvants, among them compounds with well-defined molecular targets, such as PAMPs, cytokines, and cyclic di-nucleotides [45]–[47]. For example, the TLR9-agonist CpG enhanced immune responses after vaccination against hepatitis B, anthrax, influenza, and malaria [48]–[51] and proved promising in vaccination of otherwise nonresponsive immune-compromised organisms [52]. However, many molecular mechanisms of adjuvanticity are still elusive. First insights were gained in receptors and signaling pathways involved in the recognition and processing of pathogenic factors and adjuvants in cells of the innate immune system [53]–[55]. Nevertheless, the discovered mechanisms of adjuvanticity do not translate to generally applicable strategies for rationally designed vaccines (see also [4]). Hence, to date, adjuvantation requires an additional solid theoretical background for systematic implementation in rational vaccine design.

## Antigen Delivery Systems

Delivery systems become necessary when antigens are not efficiently transported to the inductive sites or presented to the immune system. For example, rapid degradation can result in weak or virtually absent responses to otherwise immunogenic antigens. The coding sequence of an antigen can be integrated into a live virus-vector, which infects antigen-presenting cells (APCs), preferentially dendritic cells (DCs) [56], [57]. The antigen is then directly presented by MHC molecules and can be recognized by TCRs. The continuous antigen expression leads to its persistent exposure to immune cells. Recombinant viral vectors can be modified with regard to effector cell targeting, expression promoters, and the type of antigenic transgene. Lentiviral vectors with improved safety and efficiency parameters have a comparatively high capacity for encoding transgenes, high transduction efficiency, low anti-vector host immunity, low genotoxicity, and persistent gene expression [58]. They proved promising in vaccination of mice with HIV-derived antigens and in nonhuman primates with SIV-derived antigens [59], [60]. In spite of the adenoviral vaccine vector's known limited efficacy due to preexisting immunity in large populations [61], [62], it still induces protective immune responses with characteristic induction of CD8^+^ T cells in humans [63]. Recombinant adenoviral vectors derived from uncommon human serotypes, chimpanzee or human/chimpanzee chimeras can circumvent the problem of host immunity [64]–[66]. Human cytomegalovirus (hCMV) vaccine vectors are based on the ability of hCMV strains to superinfect individuals with persistent hCMV infection and immunity. Rhesus macaques developed specific CD4^+^ and CD8^+^ responses against SIV antigens delivered by a recombinant CMV vector [67], [68]. Elucidation of the molecular mechanisms leading to memory inflation during chronic hCMV infections might even lead to hCMV-based strategies to trigger life-long responses. Attenuated recombinant poxviruses are also intrinsically immunogenic, and insights in the promoted innate immune responses have accumulated [69]. The above-described vectors have considerable potential in human vaccination, especially in prime-boost regimens aimed at fine-tuning responses [70]. Different attenuated or commensal bacteria have also been successfully exploited for delivering vaccine antigens and biologicals [71]–[75].

The delivery to DCs can be achieved by coupling antigens to antibodies specific for surface molecules, such as Clec9A. This method leads to antigen uptake and activation of T and B cells [76]. Similarly, fusion proteins of HIV antigens and antibody fragments targeting the DC surface molecule DEC205 elicited potent cellular immunity in nonhuman primates [77]. The risks related to live vectors in immune-compromised individuals can be eliminated by the application of virus-like particles (VLPs) that are reduced to the structures and antigenic components necessary for delivery and immunogenicity. VLPs are able to elicit efficient humoral immune responses [78]–[80], contributing to the control of infection [78], [81]. Plasmid DNA vectors can be delivered to cells and elicit humoral responses [82], as proven by DNA vaccines against seasonal influenza in phase I trials [83]. Synthetic delivery systems, such as nanoparticles, block-copolymers, DNA nanostructures, and nanogels [84]–[86], can be loaded or coated with specific antigens and adjuvants. In addition, they can be tailored and functionalized according to specific needs (e.g., transcutaneous or mucosal delivery) [87], [88]. Trials with nanoparticle vaccines for hepatitis B, leishmaniasis, and malaria demonstrated that they enhance immune responses [87], [89], [90]. Although often developed on an empirical base, the given examples are a proof-of-principle essential to rationally design such delivery vehicles in the future.

## Immune Response Prediction

Understanding what is needed to confer protection without side effects is a prerequisite to develop a tailored intervention. To date, characterization of human responses to vaccination relies mainly on measuring antibody titers or cellular responses from peripheral blood samples. This does not allow a comprehensive analysis of responses with regard to the effector cells or mechanisms stimulated and the status in all relevant compartments for acquired immunity. Efforts to tackle this problem link the regulation of transcription or protein activity to the prediction of vaccination outcomes [91]. Recent reports suggest the potential of systems vaccinology for the analysis of gene expression profiling experiments to identify patterns or signatures linked to a desired outcome of vaccination [92]–[94]. Human studies showed correlations of gene expression profiles or protein expression patterns with immune system activation upon vaccination against yellow fever and influenza in responders and nonresponders [95]–[97]. Others characterized transcription profiles after treatment of mice or murine DCs with adjuvant molecules [98], [99]. Correlations between successful immunization or toxic events and cellular expression profiles can be predictive for a particular vaccine. However, no general unambiguous markers were identified that would allow accurate prediction of efficacy or safety for vaccines in trials (introduced, for example, in [5]).

A quite different approach to predict immune responses upon exposure to potential immunogens is realized by the *in silico* immune system simulator C-ImmSim [100]–[102]. This model features simulation of different classes of B and T lymphocytes, innate immune cells (e.g., DCs and macrophages), and different immune compartments (e.g., bone marrow, thymus and tertiary lymphoid organs). *In silico* experiments simulate primary immune responses as well as challenge with a particular antigen in different definable MHC allele backgrounds. The proof-of-principle was performed with antigens of HIV or influenza virus that simulate immunization. The simulations could indeed predict observations in humans, for example that affinity maturation and antigenic dominance evolve, and that MHC diversity can have an impact on immune defense [100]. C-ImmSim can be updated whenever improved versions of the incorporated BCR and TCR epitope prediction methods become available. Though currently not successfully applied, the simulator has potential in vaccine development by testing the immunogenicity of antigens and the potency to induce a robust immune response upon challenge with the antigen. It can be also used as a research tool to elucidate mechanisms of immune responses to fill gaps in knowledge that slow down rational design efforts.

A prevalent problem in vaccine translation is the delayed and costly transition from preclinical to clinical development due to difficulties in predicting human immune responses. Although closer to humans, primate models are associated with ethical, logistic, and financial constraints. An emerging alternative is the use of mice humanized for the immune system. Although they still need to be improved, they can be foreseen as powerful tools to predict human-specific immune responses to vaccines, as well as to investigate vaccine efficacy against pathogens with human tropism [103]–[105].

## Concluding Remarks

**Figure 1:**
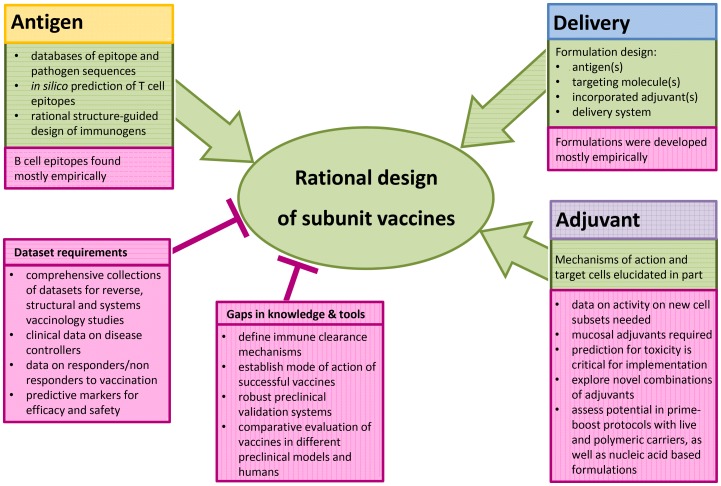
Optimizing the design for more efficient vaccines. Modern vaccinology focuses on the development of subunit vaccines to maximize efficacy and minimize risks in healthy and immune-compromised individuals. Different enabling technologies and knowledge contribute towards the rational design of formulations that would not only exhibit improved performance but also reduce the time and costs associated with preclinical and clinical development. Promising approaches/enabling factors and roadblocks are highlighted in green and pink, respectively.

In this review we elaborate on recent achievements that facilitate rational vaccine design. There are many visions on the expected impact of reverse vaccinology, epitope prediction, structural vaccinology, systems vaccinology, and personalized medicine on the rational design of effective vaccines [3], [5], [6], [106]. However, the implementation of these concepts towards the development of new and more potent vaccines requires time and considerable financial investment. Rational vaccine design will rely strongly on the availability of clinical data on individuals with different clinical forms of disease or response to vaccination to learn what is needed for protection [107]. The gaps in knowledge on the immune system's specific clearance mechanisms against many pathogens slow down the identification of the immune response that should be evoked by tailored vaccines in different population groups (Table 1). Many aspects of the host pathogen interaction and host immune status during persistent infection are also poorly understood, thereby hindering the development of therapeutic vaccines [108]. Further data from trials with empiric formulations are required to identify patterns or biomarkers that can reliably guide prediction of vaccine efficacy and safety at reasonable success rates (Figure 1). A widely accepted goal in vaccine development is the applicability to huge populations, if not all humankind. Nevertheless, there are reasons for more personalized approaches that consider specific preconditions in recipients, such as genetic background, pre-exposure to pathogens or vaccines, unique physiological background related to local culture/habits, age, and immunodeficiency.

**Table 1:** Needs and challenges for the rational design of vaccines.

Subunit Vaccine Component	Focus of Future Developments	Benefit Toward Rational Design
Antigens	Knowledge on the most effective immune response against a particular pathogen	Selection of antigens and formulations evoking those responses
	Antibody epitope database	Basis for development of computational prediction tools
	Prediction of sequences that should be excluded due to (i) risk of autoimmune responses, (ii) immune escape by antigenic drift, and (iii) responses to only selected strains or clades of the pathogen	Design of antigens capable of eliciting potent cross-reactive immune responses with minimal risk for side effects
	Continuous survey and registration of evolving pathogenic strains and clades	Improved coverage for selected antigens
	Investigation of protein/peptide degradation rules for different vaccination routes	Improved stability of designed antigens
	Extension of MHC allele–peptide complex databases, especially for MHC class II	Increased reliability of epitope prediction with already available tools
Delivery systems	Advancement of nanotechnologies	Improved synthetic delivery systems
	Investigation of mechanisms to overcome preexisting immunity or persistent virus superinfection	Maximizes potential of live vectors derived from pathogens causing common human chronic infections
	Understanding the basis for eliciting memory responses	Design of vaccines triggering long-lasting protection
	Investigation of the interface between innate and adaptive immunity	Exploitation of optimal APC targets and intrinsic adjuvant properties of the delivery system
Adjuvants	Knowledge on the most effective immune response against a particular pathogen	Selection of adjuvants facilitating those responses
	Investigation of vaccination route-dependent adjuvant effects	Optimized use of adjuvants and vaccine design
	Elucidation of molecular mechanisms of adjuvanticity	Optimizes adjuvant use and forecasts potential side effects
	Investigation of the basis of immune stimulation in different population groups	Development of personalized vaccines

Implementation of rational development concepts in vaccinology demands patience, and advances will be incremental. Realization will depend on the application of flanking logistic and regulatory measures and the awareness of the strong impact of vaccine development to solve global health problems. Funding is also required for the basic research needed to provide the basis for rationally developed vaccines. However, we expect to see the advent of new and more efficient vaccines in the coming years as a result of the implementation of this emerging knowledge and enabling technologies.
